# DNA polymorphism analysis of *Brucella *lipopolysaccharide genes reveals marked differences in O-polysaccharide biosynthetic genes between smooth and rough *Brucella *species and novel species-specific markers

**DOI:** 10.1186/1471-2180-9-92

**Published:** 2009-05-13

**Authors:** Michel S Zygmunt, José M Blasco, Jean-Jacques Letesson, Axel Cloeckaert, Ignacio Moriyón

**Affiliations:** 1 INRA, UR1282, Infectiologie Animale et Santé Publique, IASP, Nouzilly, F-37380, France; 2 Unidad de Sanidad Animal, Centro de Investigación y Tecnología Agroalimentaria (CITA) de Aragón, Gobierno de Aragón, Av Montañana, 930 50059 Zaragoza, Spain; 3 Laboratoire d'Immunologie et Microbiologie, Unité de Recherche en Biologie Moléculaire (URBM), Facultés Universitaires, Notre-Dame de la Paix (FUNDP), Namur, Belgium; 4 Departamento de Microbiología, Universidad de Navarra, Aptdo 177, 31080, Pamplona, Spain

## Abstract

**Background:**

The lipopolysaccharide is a major antigen and virulence factor of *Brucella*, an important bacterial pathogen. In smooth brucellae, lipopolysaccharide is made of lipid A-core oligosaccharide and N-formylperosamine O-polysaccharide. *B. ovis *and *B. canis *(rough species) lack the O-polysaccharide.

**Results:**

The polymorphism of O-polysaccharide genes *wbkE, manA*_*O*-*Ag*_, *manB*_*O*-*Ag*_, *manC*_*O*-*Ag*_, *wbkF and wbkD*) and *wbo *(*wboA *and *wboB*), and core genes *manB*_*core *_and wa** was analyzed. Although most genes were highly conserved, species- and biovar-specific restriction patterns were found. There were no significant differences in putative N-formylperosamyl transferase genes, suggesting that *Brucella *A and M serotypes are not related to specific genes. In *B. pinnipedialis *and *B. ceti *(both smooth), *manB*_*O*-*Ag *_carried an IS*711*, confirming its dispensability for perosamine synthesis. Significant differences between smooth and rough species were found in *wbkF *and *wbkD*, two adjacent genes putatively related to bactoprenol priming for O-polysaccharide polymerization. *B. ovis wbkF *carried a frame-shift and *B. canis *had a long deletion partially encompassing both genes. In smooth brucellae, this region contains two direct repeats suggesting the deletion mechanism.

**Conclusion:**

The results define species and biovar markers, confirm the dispensability of *manB*_*O*-*Ag *_for O-polysaccharide synthesis and contribute to explain the lipopolysaccharide structure of rough and smooth *Brucella *species.

## Background

The members of the genus *Brucella *are gram-negative bacteria that cause brucellosis, a zoonotic disease of great importance worldwide. Currently, several *Brucella *species are recognized [1]. *B. abortus*, *B. melitensis*, *B. suis*, *B. neotomae, B. ovis*, and *B. canis *have been known for a long time and are traditionally distinguished according to their preferential host, biochemical tests and cell surface characteristics [2]. In addition, *Brucella *strains isolated from cetaceans and pinnipeds during the last fifteen years have been grouped into *B. ceti *and *B. pinnipedialis*, [3]. Very recently, some *Brucella *strains have been isolated from the common vole and a new species, *B. microti*, proposed [4]. *B. abortus*, *B. melitensis *and *B. suis *have been classically subdivided into biovars according to H_2_S production, CO_2_-dependence, dye sensitivity and distribution of the A and M epitopes (see below) [2]. However, because these tests are difficult to standardize, molecular markers have been investigated [5-9].

**Figure 1:**
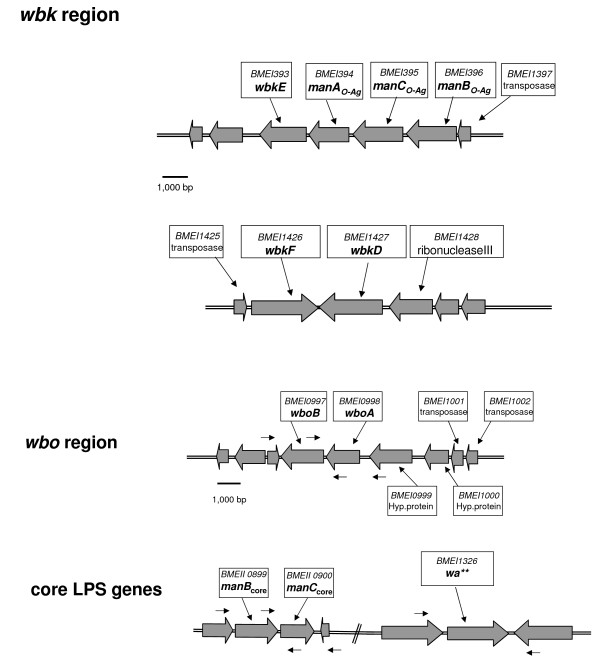
**Regions and genes encoding LPS biosynthetic enzymes in *B. melitensis *16 M Region *wbk *contains genes coding for: (i), enzymes necessary for N-formylperosamine synthesis (*gmd, per, wbkC*); (ii), two O-PS glycosyltransferase (*wbkE, wbkA*); (iii), the ABC transporter (*wzm, wzt*); (iv) the epimerase/dehydratase necessary for the synthesis of an N-acetylaminosugar (*wbkD*); and (v), the polyisoprenyl-phosphate N-acetylhexosamine-1-phosphate transferase enzyme that primes bactoprenol (*wbkF*)**. Genes *manA*_*O*-*Ag*_, *manB*_*O*-*Ag*_, *manC*_*O*-*Ag *_could be involved in the synthesis of mannose, the perosamine precursor. Restriction sites: A, *Alu*I; AvI, *Ava*I; Av, *Ava*II; B, *Bgl*I; Bg, *Bgl*II; C, *Cla*I; E, *Eco*RI; EV, *Eco*RV; H, *Hind*III; Ha, *Hae*II; Hf, *Hinf*I; P, *Pst*I; Pv, *Pvu*II; S, *Sau*3A; Sa, *SaI*I; St, *Sty*I.

Wild type *B. melitensis*, *B. abortus*, *B. suis*, *B. neotomae*, *B. ceti, B. pinnipedialis *and *B. microti *express a smooth (S)-type lipopolysaccharide (LPS) formed by an O-polysaccharide connected to a core oligosaccharide which, in turn, is linked to lipid A, the section embedded into the outer membrane. However, both *B. ovis *and *B. canis *lack the O-polysaccharide and, accordingly, their LPS is termed rough (R) (R-LPS). *Brucella *LPS is of great interest not only because of these species differences but also because it is the foremost diagnostic antigen and a major virulence factor [10]. Despite this, the structure and genetics of *Brucella *LPS is only partially understood. The O-polysaccharide is a homopolymer of N-formyl-perosamine in α (1–2) or in α (1–2) plus α (1–3) linkages [11], and these variations relate to the main serovars in *Brucella *S species (A dominant, related to the α (1–2) linkage; M dominant [α (1–2) plus α (1–3) in a 4:1 proportion]; or A = M [α (1–2) plus α (1–3) in a > 4:1 proportion]). Previous studies in *B. melitensis *16 M and H38 (both biovar 1) have identified two genetic regions involved in O-polysaccharide synthesis and translocation (Figure 1)(reviewed in [12]). Region *wbo *encodes two putative glycosyltransferases (*wboA *and *wboB*) and region *wbk *contains the genes putatively involved in perosamine synthesis (*gmd *[GDP-mannose 4, 6 dehydratase] and *per *[perosamine synthetase]), its formylation (*wbkC*) and polymerization (glycosyltransferases) (*wbkA *and *wbkE*), as well as those for bactoprenol priming (*wbkD *and *wbkF*) and O-PS translocation (*wzm *and *wzt*). In addition, *wbk *contains genes (*manA*_*O*-*Ag*_, *manB*_*O*-*Ag*_, *manC*_*O*-*Ag*_) which may code for the enzymes that furnish mannose, the perosamine precursor. Intriguingly, *wbkB *and *manB*_*O*-*Ag *_do not generate R phenotypes upon disruption [12,13], and *B. ovis *and *B. canis *carry *wbk *genes despite the absence of the O-polysaccharide [14]. Much less is known on the *Brucella *core oligosaccharide. Reportedly, it contains 2-keto, 3-deoxyoctulosonic acid, mannose, glucose, glucosamine and quinovosamine [12,15] but the structure is unknown. Thus far, only three genes have been proved to be involved in core synthesis: *pgm *(phosphoglucomutase, a general biosynthetic function), *manB*_*core *_(mannose synthesis) and wa** (putative glycosyltransferase) [12]. Obviously, genetic analysis encompassing a variety of strains could shed light on the differences behind the phenotypes of S and R species, confirm or rule out a role for known genes, and identify differences that could serve as serovar or biovar markers. With these aims, *wbkE*, *manA*_*O*-*Ag*_, *manB*_*O*-*Ag*_, *manC*_*O*-*Ag*_, wbkF, wkdD, wboA, wboB, wa** and *manB*_*core *_were analyzed for polymorphism in the classical *Brucella *spp., *B. ceti*, and *B. pinnipedialis*.

## Results

### LPS genes in *Brucella *spp. and biovars

Figure 1 shows the organization of LPS genes in *B. melitensis *16 M [12]. PCR amplification of *wbkE*, *manB*_*O*-*Ag*_, *manA*_*O*-*Ag*_, *manC*_*O*-*Ag*_, *wkdD, wbkF, wboA *and *wboB*, wa** and *manB*_*core *_was conducted on representative strains of each of the *Brucella *species included in this study and their biovars with attention to the LPS characteristics (i.e. S versus R; and A dominant, M dominant, or A = M for the S-LPS). Except for *wboA *and *wboB *in *B. ovis*, all genes were successfully amplified in the strains of all *Brucella *species and biovars tested. These results confirm the absence of the *wbo *region in *B. ovis *[16,17]. They also suggest that conservation of *wbk *extends beyond those genes (*wbkA *to *wbkC*) examined in a previous work [14] and that wa** and *manB*_*core *_were are also conserved in the genus. Further analyses were then conducted to examine these possibilities.

### Gene polymorphism in *wbk*

#### wbkE

For all strains, the *wbkE *PCR-amplified product displayed the same *Eco*RV, *Hinf*I, *Pst*I and *Pvu*II RFLP patterns. Although *B. melitensis *63/9 biovar 2 showed a different *Sty*I pattern, only one of eight additional *B. melitensis *biovar 2 strains tested showed this *Sty*I pattern (data not shown).

#### manA_*O*-*Ag*_

**Figure 2:**
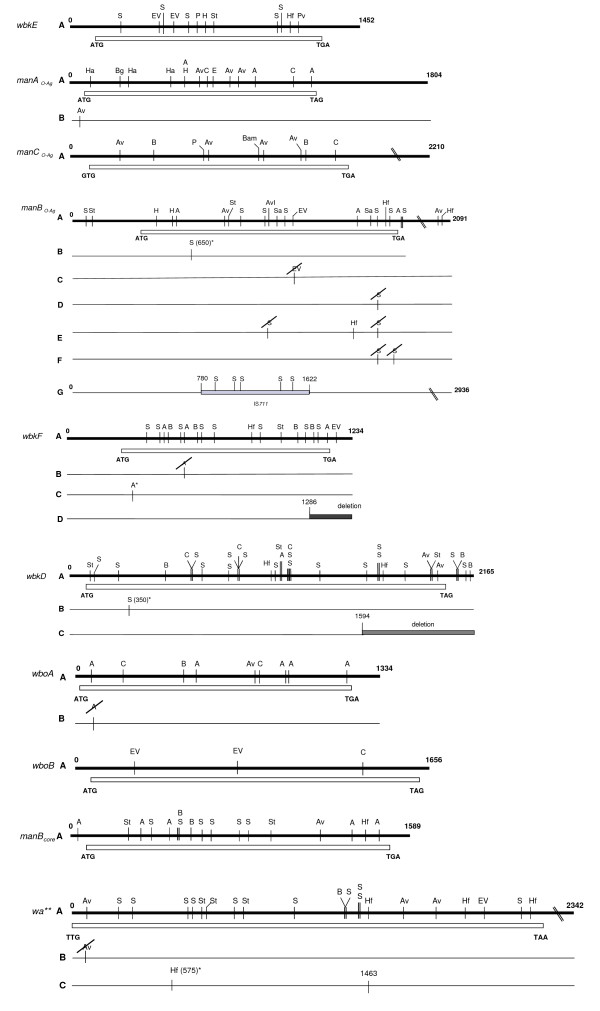
**Restriction maps of the core- and O-polysaccharide genes with the restriction enzymes used**. For each gene, restriction map A corresponds to that deduced from the nucleotide sequence of *B. melitensis *16 M. Only differences compared to the nucleotide sequences of *B. melitensis *16 M are indicated in restriction maps B, C, D, E, F and G. The restriction patterns A, B, C, D, E, F and G are further indicated in Table 1 for each gene and for each *Brucella *strain studied. Additional sites and their most probable location according to restriction patterns are indicated by the restriction name (e.g. Hf) and by the position name and an asterisk.

**Figure 3:**
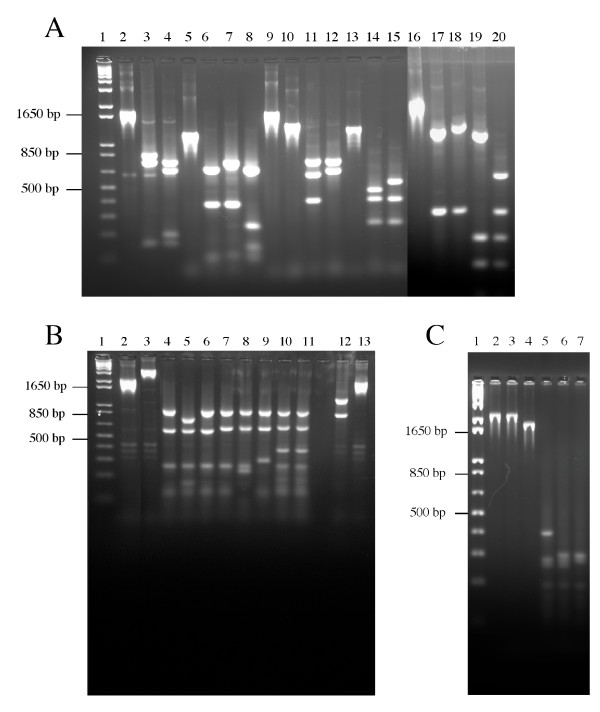
PCR-RFLP analysis of *Brucella *LPS genes *manA*_*O*-*Ag*_, *manB*_*O*-*Ag*_, *wbkD*, *wbkF*, *wboA *and wa**. Panel A. Lanes: 1, molecular size markers; 2, *manA*_*O*-*Ag *_from *B. melitensis *16 M uncut; 3, *manA*_*O*-*Ag *_from *B. melitensis *16 M cut by *Ava*II; 4, *manA*_*O*-*Ag *_from *B. neotomae *cut by *Ava*II; 5, *wbkF *from *B. melitensis *16 M uncut; 6, *wbkF *from *B. melitensis *16 M cut by *Alu*I; 7, *wbkF *from *B. melitensis *bv2 cut by *Alu*I; 8, *wbkF *from *B. abortus *bv2 cut by *Alu*I; 9, *wbkF*2* from *B. melitensis *16 M uncut; 10, *wbkF*2* from *B. canis *uncut; 11, *wbkF*2* from *B. melitensis *16 M cut by *EcoR*V; 12, *wbkF*2* from *B. canis *cut by *EcoR*V; 13, *wboA *from *B. melitensis *16 M uncut; 14, *wboA *from *B. melitensis *16 M cut by *Alu*I; 15, *wboA *from *B. abortus *cut by *Alu*I; 16, wa** from *B. melitensis *16 M uncut; 17, wa** from *B. melitensis *16 M cut by *Ava*II; 18, wa** from *B. suis *bv2 cut by *Ava*II; 19, wa** from *B. melitensis *16 M cut by *Hinf*I; 20, wa** from *B. ovis *cut by *Hinf*I. Panel B. Lanes: 1, molecular size markers; 2, *manB*_*O*-*Ag *_from *B. melitensis *16 M uncut; 3, *manB*_*O*-*Ag *_from *B. pinnipedialis *uncut; 4, *manB*_*O*-*Ag *_from *B. melitensis *16 M cut by *Sau*3A; 5, *manB*_*O*-*Ag *_from *B. melitensis *bv2 cut by *Sau*3A; 6, *manB*_*O*-*Ag *_from *B. abortus *cut by *Sau*3A; 7, *manB*_*O*-*Ag *_from *B. suis *cut by *Sau*3A; 8, *manB*_*O*-*Ag *_from *B. suis *bv2 cut by *Sau*3A; 9, *manB*_*O*-*Ag *_from *B. ovis *cut by *Sau*3A; 10, *manB*_*O*-*Ag *_from *B. pinnipedialis *cut by *Sau*3A; 11, *manB*_*O*-*Ag*_from *B. ceti *cut by *Sau*3A; 12, *manB*_*O*-*Ag*_from *B. melitensis *16 M cut by *Eco*RV; 13, *manB*_*O*-*Ag *_from *B. abortus *cut by *Eco*RV. Panel C. Lanes: 1, molecular size markers; 2, *wbkD *from *B. melitensis *16 M uncut; 3, *wbkD *from *B. abortus *uncut; 4, *wbkD *from *B. canis *uncut; 5, *wbkD *from *B. melitensis *16 M cut by *Sau*3A; 6, *wbkD *from *B. abortus *cut by *Sau*3A; 7, *wbkD *from *B. canis *cut by *Sau*3A.

*B. neotomae *had a distinct *manA*_*O*-*Ag *_restriction pattern consisting of an additional *Ava*II site (Figures 2 and 3, Table 1). Moreover, *in silico *analysis showed a specific profile for *B. ovis *consisting of a nucleotide substitution (GAA to GGA) at position 497 which modified the ManA C-terminal sequence at amino acid 165 (not shown). Also, a single nucleotide deletion (CAAT to CA-T) was detected at position 738; this frame shift leads to a change in amino acid sequence after position 246. Nucleotide sequence of PCR products from several strains confirmed the deletion in *manA*_*O*-*Ag *_as characteristic of *B. ovis *(not shown).

**Table 1:** *Brucella *strains used in this study.

								O-chain biosynthetic gene restriction patterns:							
								*wbk* region				*wb* region			
Species	Biovar	Serotype	Strain	Host/ source	Geographic origin	*wbkE*	*manA* *O-Ag*	*manC* *O-Ag*	*manB* *O-Ag*	*wbkF*	*wbkD*	*wboA*	*wboB*	*manB* *core*	wa**
Terrestrial mammal:															
*B. melitensis*	1	M	16 M (ATCC 23456; BCCN R1)	Goat	United States	A	A	A	A	A	A	A	A	A	A
	2	A	63/9 (ATCC 23457; BCCN R2)	Goat	Turkey	A	A	A	B	B	A	A	A	A	A
	3	AM	Ether (ATCC 23458; BCCN R3)	Goat	Italy	A	A	A	B	A	A	A	A	A	A

*B. abortus*	1	A	544 (ATCC 23448; BCCN R4)	Cattle	England	A	A	A	C	A	B	B	A	A	A
	2	A	86/8/59 (ATCC 23449; BCCN R5)	Cattle	England	A	A	A	C	C	B	B	A	A	A
	3	A	Tulya (ATCC 23450; BCCN R6)	Human	Uganda	A	A	A	A	A	B	B	A	A	A
	4	M	292 (ATCC 23451; BCCN R7)	Cattle	England	A	A	A	C	A	B	B	A	A	A
	5	M	B3196 (ATCC 23452; BCCN R8)	Cattle	England	A	A	A	C	A	B	B	A	A	A
	6	A	870 (ATCC 23453; BCCN R9)	Cattle	Africa	A	A	A	C	A	B	B	A	A	A
	9	M	C68 (ATCC 23455; BCCN R11)	Cattle	England	A	A	A	C	A	B	B	A	A	A
		R	45/20 (BCCN V2)	Cattle	England	A	A	A	C	C	B	B	A	A	A

*B. suis*	1	A	1330 (ATCC 23444; BCCN R12)	Swine	United States	A	A	A	D	A	B	A	A	A	A
	2	A	Thomsen (ATCC 23445; BCCN R13)	Swine	Denmark	A	A	A	E	A	B	A	A	A	B
	3	A	686 (ATCC 23446; BCCN R14)	Swine	United States	A	A	A	D	A	B	A	A	A	A
	4	AM	40 (ATCC 23447; BCCN R15)	Reindeer	Russia	A	A	A	D	A	B	A	A	A	A
	5	M	513 (BCCN R21)	Wild rodent	Russia	A	A	A	D	A	B	A	A	A	A

*B. ovis*			63/290 (ATCC 25840; BCCN R17)	Sheep	Africa	A	A	A	F	A	B	NA	NA	A	C
			Reo 198 (BCCN R22)	Sheep	United States	A	A	A	F	A	B	NA	NA	A	C
			BCCN 76–250	Sheep	France	A	A	A	F	A	B	NA	NA	A	C

*B. canis*			RM6/66 (ATCC 23365; BCCN R18)	Dog	United States	A	A	A	D	D	C	A	A	A	A
			D519 (BCCN C1)	Dog	Madagascar	A	A	A	D	D	C	A	A	A	A
			BCCN 87.65	Dog	Canada	A	A	A	D	D	C	A	A	A	A

*B. neotomae*		A	5K33 (ATCC 23459; BCCN R16)	Desert rat	United States	A	B	A	D	A	A	A	A	A	A

Marine mammal:															
*B. pinnipedialis*		A	B2/94	Common seal	Scotland	A	A	A	G	A	A	A	A	A	A

*B. ceti*		A	B1/94	Porpoise	Scotland	A	A	A	G	A	A	A	A	A	A

^a ^ATCC, American Type Culture Collection; BCCN, Brucella Culture Collection, Nouzilly, France.

NA: Not Amplified

#### manC_*O*-*Ag*_

Despite the use of several endonucleases (*Bam*HI, *Ava*I, *Ava*II, *Bgl*I, *Cla*I, *Pst*I), *manC*_*O*-*Ag *_restriction patterns were identical in all *Brucella *strains (Figure 2, Table 1). Therefore, no polymorphism was observed by this method.

#### manB_*O*-*Ag*_

*B. melitensis *16 M (biovar 1) and *B. abortus *Tulya (biovar 3) presented a similar *manB*_*O*-*Ag *_restriction pattern (pattern A), and *B. melitensis *biovars 2 and 3 showed a *Sau*3A site absent in other strains (pattern B). All *B. abortus *(except *B. abortus *Tulya (biovar 3)) strains tested showed a specific pattern characterized by the absence of the *Eco*RV site at position 1238 (pattern C). *B. suis *biovars 1, 3, 4 and 5, *B. canis *and *B. neotomae *formed a separate group (pattern C) on the basis of the *Sau*3A restriction patterns of this gene. *B. ovis *shared this pattern only partially because it lacked one more *Sau*3A site (pattern F). *B. suis *biovar 2 strains lacked the *manB*_*O*-*Ag *_*Sau*3A site and showed an additional *Hinf*I site in this gene (pattern E). When this gene was amplified (primers *manB*-A and *manB*-B; (Table 2) from *B. ovis *63/290, sequenced, and aligned with the homologous genes of *B. melitensis *biovar 1, *B. abortus *biovar 1, and *B. suis *biovar 1, polymorphism in both sequence and length was detected. As compared to *B. melitensis *biovar 1 and *B. abortus *biovar 1, two more nucleotides were found at position 1265–1266 in *B. suis *biovar 1 and *B. ovis *which should lead to a modification of C-terminal sequence of the protein (not shown). All strains isolated from marine mammals yielded restriction *manB*_*O*-*Ag *_patterns very different from those of the six classical species (pattern G, Table 1) as well as a larger PCR product (2,933 bp and 2,091 bp, respectively) (Figure 3). Sequencing of the PCR product of three strains (B2/94, B1/94 and B14/94) revealed an IS*711 *element (842 bp) inserted into the gene (from position 780 to 1622) (Figure 2), and this insertion was confirmed by PCR in 82 additional marine mammal strains (not shown).

**Table 2:** DNA Primers used

Target DNA	Primer name	Sequence (5'-3')	Amplicon size
*manAO-Ag*	manA-A	CATCACCATCGTTCAGAGCA	1804 bp
	manA-B	GCCAGGGGAAATGATAATGA	
*manBO-Ag*	manB-A	GTTGGCAGAAGTTGGCATCG	2091 bp
	manB-B	CTAATGCCTGTTCCGCCACC	
*manCO-Ag*	manC-A	TTGAAGACTGGTTTATTGCG	2210 bp
	manC-B	GCAAGACTGCCATAGAAACC	
*wbkE*	wbkE-A	CCGCAAACTGAATGGATAAA	2452 bp
	wbkE-B	GCAACTGTCAGGTCTGGTGC	
*wbkF*	wecA-A	GCGGAGGAATGGACAAGGAC	1234 bp
	wecA-B	AGGAAAGCCTGGCGGTACTG	
*wbkD*	wbkD-A	TGGCTGGAGTGTGCCGAAAG	2165 bp
	wbkD-B	ACGGTTGCTGGTGCTTGTGG	
wa**	wa**-A	CATCACGCATAATGACACCG	2342 bp
	wa**-B	TGCTTTTGACAAGCTCGTCG	
*manBcore*	manBcore-A	CCAGCCGACGATTGAACTGG	1589 bp
	manBcore-B	AAGCCTTGAACCCGATCCCC	
*wboA*	wboA-A	TCTGCATAACGGTCCTTGCC	1334 bp
	wboA-B	GCTTTTACGGCAACAAGTCC	
*wboB*	wboB-A	CTTGGGATGCGAAACTACCG	1656 bp
	wboB-B	GCTCACGCTTCCGAATACTG	

#### *wbkD *and *wbkF*

**Figure 4:**
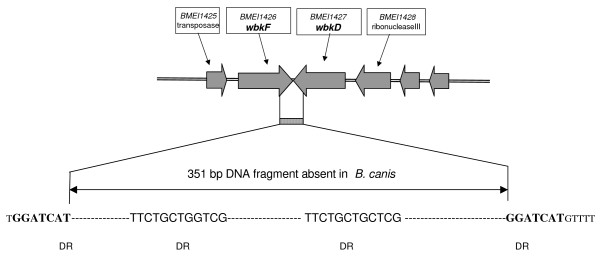
**The *B. melitensis *16 M chromosome I region absent in *B. canis *and the adjacent DNA**. The two 7 bp direct repeats located in *B. melitensis *16 M at both sides of the fragment absent in *B. canis *are in bold.

**Figure 5:**
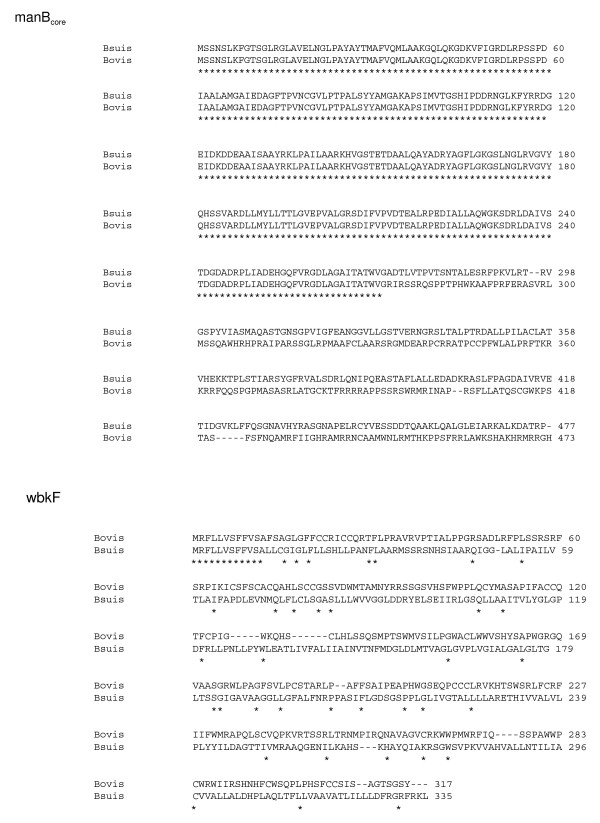
**Comparison of the *B. suis *ManB_core _and WbkF with the corresponding *B. ovis *proteins**. Conserved amino acids are indicated by stars. The alignment was performed using the Clustal W program.

The *wbkD *PCR product was tested with *Hinf*I, *Ava*II, *Sau*3A, *Bgl*I, *Cla*I and *Sty*I, but a very low degree of DNA polymorphism was observed (Figures 2 and 3, and Table 1). For *B. melitensis, B. neotomae *and all marine mammal strains, all strains showed the same *Sau*3A pattern. An additional *Sau*3A site was observed for all *B. abortus, B. suis *and *B. ovis *strains (pattern B). Interestingly, the *B. canis *product showed a reduced size of around 400 bp and, therefore, yielded species specific restriction patterns(Figures 2 and 3). This result indicated the existence of a deletion in *B. canis wbkD *(see below). The *wbkF *PCR product showed also a low degree of polymorphism when tested with *Eco*RV, *Hae*II, HinfI, *Alu*I, *Sau*3A and *Sty*I (Figures 2 and 3, and Table 1). One pattern, however, was specific for *B. melitensis *biovar 2 which lacked an *Alu*I site, and a distinct pattern for two *B. abortus *biovar 2 and 45/20, was also observed with *Alu*I site. Remarkably, no amplification was obtained for *B. canis*, suggesting that the sequence of the *wbkF*-B primer corresponded to a deletion extending from the adjacent *wbkD *gene (see above). In fact, when the appropriate primer was used, the *wbkF *PCR product showed a reduced size of about 400 bp. To examine this point further, the *wbkF-wbkD *locus was amplified and sequenced in *B. melitensis, B. ovis *and *B. canis*. The sequences showed a 351 bp deletion in *B. canis *extending from *wbkD *nucleotide 1594 (in BMEI 1426) to *wbkF *nucleotide 918 (in BMEI 1427) (Figure 3 and 4) as confirmed by the genome sequence of *B. canis *RM 6/66 (ATCC 23365) (Genbank accession # CP000872 and CP000873). Moreover, as compared to their homologs in *B. melitensis*, *B. abortus *and *B. suis*, gene *wbkF *of *B. ovis *showed a single nucleotide deletion at position 35. This frame shift mutation necessarily leads to an extensive modification of cognate protein (Figure 5).

### Gene polymorphism in *wboA*

A low degree of DNA polymorphism was observed in *wboA*. However, one pattern was specific of *B. abortus *since all strain testedlacked an *Alu*I site. As described above, no amplification was observed for any *B. ovis *strain. This confirms [16,17] that absence of *wboA *(and *wboB*) is a *B. ovis *species-specific marker.

### Polymorphism in core LPS genes

Despite using six restriction enzymes, all brucellae displayed the same RFLP pattern for the *manB*_*core *_amplicon. *In silico*, the four genomes available showed low polymorphism. A single nucleotide deletion at position 812 was detected in *B. ovis*, which should modify the C-terminal sequence of the protein (Figure 5). Similarly, a low degree of polymorphism was observed in wa**. With the exception of *B. suis *biovar 2, one *Pst*I pattern was specific of *B. suis*. Biovar 2 also lacked an *Ava*II site, which could be considered as a biovar marker. With *Hinf*1, a pattern was specific of *B. ovis *(Figure 2, Table 1).

## Discussion

Despite the high DNA homology of brucellae, gene polymorphism and species- and biovar-specific markers have been consistently found. Concerning outer membrane molecules, both have been found in genes of proteins [16,18,19] but not in the LPS genes examined, all of the *wbk *region (*wbkA, gmd, per, wzm, wzt, wbkB*, and *wbkC*). Interestingly, these O-polysaccharide genes were found to be highly conserved not only in the classical S *Brucella *species and biovars but also in *B. ovis *and *B. canis*, the two species that lack the O-polysaccharide [14]. Therefore, an implication of these observations is that the R phenotype of *B. ovis *and *B. canis *cannot be explained by the absence of any of those seven *wbk *genes. More recently, the *wbk *region has been extended to include *wbkE*, *manA*_*O*-*Ag*_, *manB*_*O*-*Ag*_, *manC*_*O*-*Ag*_, *wbkF*, and *wkdD *[12]. The present study includes an analysis of some of these genes and the results not only show the existence of specific markers but, more important, they also improve our understanding of the genetics-structure relationship in *Brucella *LPS. Concerning the O-polysaccharide, the results are relevant to interpret the variations in O-polysaccharide linkages of S *Brucella *and add further weight to our previous finding (12) that the putative mannose genes in *wbk *are not essential for perosamine synthesis. Furthermore, they help to explain the differences existing between S and R *Brucella *species.

Despite extensive transposon mutagenesis searches, only four putative glycosyltransferase genes have been implicated in N-formylperosamine polymerization in *Brucella*: *wbkA*, *wbkE*, *wboA *and *wboB*. As mentioned above, *wbkA *is conserved in classical *Brucella *species [14], and the results reported here show that *wboA*, *wboB *and *wbkE *are similarly present in S *B. melitensis*, *B. abortus*, *B. suis*, *B. pinnipedialis *and *B. ceti*. Moreover, these genes displayed low polymorphism, no matter the A or M serotype. It has to be noted that the consensus sequences of glycosyltransferases are conspicuous enough to make unlikely the existence of O-polysaccharide transferases other than *wboA*, *wboB, wbkA *and *wbkE*, and that, although the α (1–3) linkage relates to the M serotype, there is evidence showing that at least some A dominant strains generate a very small proportion (i.e. 2%) of α (1–3) linkages [20]. In keeping with this, it has been observed that strain RB51 (a *wboA *mutant of the A-dominant *B. abortus *2308 S strain [21]) generates small amounts of atypical M-type polysaccharides [22]. All this evidence suggests that, rather than the presence of a α (1–3)-specific transferases in the M serotype, there are subtle variations in the expression of *wboB, wbkA *or *wbkE*, or in the activity of the corresponding glycosyltransferases that lead to the increase in α (1–3) linkages typical of the M and A = M serotypes.

A surprising feature of the *wbk *is the presence of genes that are not essential for O-polysaccharide synthesis. Godfroid et al. [13] analyzed the functions of the ORFs between BMEI1404 (*wbkA*, encoding a putative mannosyltransferase [perosaminyltransferase since mannose and perosamine are related]) and BMEI1418 (*wbkC*, encoding a putative formyltransferase) and found that disruption of ORF BMEI1417 (*wbkB*) generated no R phenotype. Later, it was found that the genome of *B. melitensis *contains three putative mannose synthesis genes (ORFs BMEI1394 to BMEI1396) adjacent to *wbkA*. Because mannose is the direct precursor of perosamine and O-polysaccharide genes usually cluster together, Monreal et al. [23] proposed the names of *manA*_*O*-*Ag*_, *manB*_*O*-*Ag*_, *manC*_*O*-*Ag *_for BMEI1394 to BMEI1396, and their assignment to *wbk *is supported by the finding by González et al. [12] that disruption of ORF BME1393 (*wbkE*) blocks O-polysaccharide synthesis. The latter authors provided proof that at least *manB*_*O*-*Ag*_, is dispensable for perosamine synthesis but also pointed out that the existence of *manB*_*core*_-*manC*_*core *_(ORFs BMEII0900 and BMEII0899) preclude to rule out any role for the *wbk *putative mannose synthesis genes since there could be internal complementation [12]. All these results are fully consistent with the observation that, although *manB*_*O*-*Ag *_is disrupted by IS711 in *B. pinnipedialis *and *B. ceti*, these two species keep the S phenotype. The *wbk *region has features suggestive of horizontal acquisition [14] whereas *manB*_*core *_(and *manC*_*core*_) are *Brucella *older genes necessary for the synthesis of the LPS core oligosaccharide [23,24]. Accordingly, a drift to dysfunction of the *wbk man *genes may have been made possible by the redundancy created after horizontal acquisition of *wbk*, and the similarity in this regard between *B. ceti *and *B. pinnipedialis *suggests a common ancestor.

The results of this research also shed additional light on the genetic basis behind the R phenotype of *B. ovis *and *B. canis*. Previous work has shown a large deletion in *B. ovis *that encompasses *wboA *and *wboB *[16,17]. The present work confirms the absence of these two putative perosaminyltraneferase genes in *B. ovis*, an absence that can account by itself for the lack of O-polysaccharide in this species [12,25]. To this evidence, the present work adds the nucleotide deletion detected in *B. ovis wbkF*. Indeed, the frame-shift thus created predicts a very modified protein. Presumably, WbkF is involved in catalyzing the transfer of an acetylated aminosugar to undecaprenylphosphate, thus priming this carrier for O-chain polymerization. The N-terminal region of the *E. coli *WbkF homologue was found to be necessary for this function [26] and, therefore, it seems likely that the frame-shift in *B. ovis wbkF *produces a non-functional protein, thus explaining in part the R phenotype of this species. Other changes detected in several *B. ovis *LPS genes do not have this dramatic effect. As discussed above, the *man wbk *genes are dispensable and, therefore, the nucleotide substitution and frame shift detected in *B. ovis manA*_*O*-*Ag *_do not contribute to the R phenotype. Since disruption of *manB*_*core *_generates a deep R-LPS [24,24], the presence of two more nucleotides in the sequence of *B. ovis manB*_*core *_was interesting. However, this deletion modified only the C-terminal sequence (5 last amino-acids) of the protein making unlikely a change severe enough to contribute to the R phenotype. In support of this interpretation, *B. ovis *R-LPS is not deeply truncated like that of *manB*_*core*_mutants. Moreover, the same two nucleotide addition was detected in *B. suis*, and it is known that a functional *manB*_*core *_is required for the synthesis of S-LPS in this species [27].

A DNA deletion of 351 bp. including 3' end of *wbkF *and 3' end of *wbkD *was detected in *B. canis*, which might have occurred by a slipped mispairing mechanism (a direct repeat sequence of 7 bp «GGATCAT» is present at both sides of the deleted sequence in the other *Brucella *species (Figure 5). It is clear that this deletion has profound consequences in the synthesis of LPS. We have discussed above the essential role of *wbkF *in O-polysaccharide synthesis, and *wbkD *seems involved in the synthesis of quinovosamine, a sugar that is possibly linking the *Brucella *O-polysaccharide to the R-LPS [12]. This double mutation clearly explains the R phenotype of *B. canis *and is consistent with the absence of quinovosamine in this species [28].

## Conclusion

The analyses carried out suggest new hypothesis to study the genetics of *Brucella *O-polysaccharide serotypes and provide evidence on both the dispensability of some *wbk *genes which is consistent with their horizontal acquisition. They also confirm the essential role of *wbkD *and *wbkF *in O-polysaccharide synthesis and, at the same time, contribute to understand the R phenotype of *B. ovis *and *B. canis*. Finally, they provide several biovar and species specific markers that can be used to design the corresponding molecular typing tools.

## Methods

### *Brucella *strains

The strains (Table 1) were maintained freeze-dried in the INRA *Brucella *Culture Collection, Nouzilly (BCCN), France. For routine use, they were grown on tryptic soy agar (Difco)-0.1% (w/v) yeast extract (Difco). Fastidious strains (*B. abortus *biovar 2 and *B. ovis*) were grown on the same medium supplemented with 5% sterile horse serum (Gibco BRL). All strains were checked for purity, and species and biovar confirmed by standard procedures [2].

### DNA preparation

Bacteria were cultured at 37°C for 24 h, suspended in 3 ml sterile distilled water, harvested (2000 × *g*, 10 minutes) and resuspended in 567 μl of 50 mM Tris, 50 mM EDTA, 100 mM NaCl (pH 8.0). Then, 30 μl of 10% (w/v) SDS and 3 μl of 2% (w/v) proteinase K were added, the mixture was held at 37°C for 1 h and extracted twice with phenol-chloroform. Nucleic acids in the aqueous phase were precipitated with two volumes of cold ethanol, dissolved in 100 μl of 10 mM Tris, 1 mM EDTA (pH 8.0) and the amount of DNA estimated by electrophoresis on 0.8% agarose gels using appropriate DNA solutions as the standards.

### Polymerase chain reaction-restriction fragment length polymorphism (PCR-RFLP)

The 20-mer primers were selected to amplify *manB*_*O*-*Ag*_, *manA*_*O*-*Ag*_, *manC*_*O*-*Ag*_, *wbkF, wkdD, wbkE*, *wboA *and *wboB*, wa* and *manB*_*core *_according to the *B. melitensis *16 M genome sequence (Genbank accession numbers AE008917 and AE008918) (Table 2). Amplification mixtures were prepared in 100 μl volumes containing 10 mM Tris-HCl (pH 9.0), 50 mM KCl, 1.5 mM MgCl_2_, 0.1% Triton X-100, 0.2 mg ml^-1 ^gelatin (1 × PCR buffer; Appligene), 200 μM each deoxynucleoside triphosphate, 1 μM each primer, 100 ng of genomic DNA, and 2.5 U of *Taq *DNA polymerase (Appligene). Amplification was performed in a GeneAmp PCR System 9600 thermocycler (Perkin Elmer) as follows: cycle 1, 94°C for 5 minutes (denaturation); the next 30 cycles, 58°C for 30 s (annealing), 70°C for 30 s (extension) and 94°C for 30 s (denaturation); the last cycle, 58°C for 30 s (annealing) and 70°C for 10 minutes (extension). For PCR-RFLP, *Alu*I, *Ava*I, *Ava*II, *Bam*HI, *Bgl*I, *Bgl*II, *Cla*I, *Eco*RI, *Eco*RV, *Hind*III, *Hae*II, *Hinf*I, *Pst*I, *Pvu*II, *Sau*3A, *SaI*I, *Sty*I were used. The restriction enzymes were chosen according to the *B. melitensis *16 M genomic sequences of the above-listed genes.

### 2.4. Nuceotide sequence and data analysis

PCR products of the expected sizes were purified from 1% agarose gels (Invitrogen) with a QIAquick gel extraction kit (Qiagen GmbH, Hilden, Germany), cloned into pGEM-T Easy vector (Promega, Madison, Wis.), and transformed into competent JM109 *Escherichia coli *cells (Promega). The transformants were selected with ampicillin, and recombinants were selected by blue-white differentiation. Plasmids were isolated from several clones with a Qiagen Plasmid Mini kit. To check for the presence of the correct insert, plasmids were digested with EcoRI and the restriction products were separated on 1% agarose gels. Nucleotide sequencing of clone was performed by automated cycle sequencing with Big Dye terminators (ABI 377XL; PE Applied Biosystems, Foster City, Calif.) and primers RP (reverse primer) and UP (universal primer M13-20). Multiple DNA and amino acid sequence alignments were performed with CLUSTAL W http://www2.ebi.ac.uk/clustalw/.

### Nucleotide sequence accession numbers

The *Brucella *nucleotide sequences determined in this work have been deposited in the GenBank/EMBL/DDBJ databases under the following accession numbers: FJ376556, FJ 376557 for the *manB*_*O*-*Ag *_gene of *B. pinnipedialis *B2/94 and *B. ceti *B1/94.

## Authors' contributions

MSZ, IM and AC conceived the study. MSZ designed and performed the experimental work. All authors analyzed the data. MSZ wrote the manuscript. IM, and AC helped to draft the manuscript. All authors read, corrected and approved the final manuscript.
